# Study of Sex Differences in the Whole Brain White Matter Using Diffusion MRI Tractography and Suprathreshold Fiber Cluster Statistics

**DOI:** 10.1101/2025.09.27.679006

**Published:** 2025-09-28

**Authors:** Fan Zhang, Jarrett Rushmore, Yijie Li, Suheyla Cetin-Karayumak, Yang Song, Weidong Cai, Carl-Fredrik Westin, James J. Levitt, Nikos Makris, Yogesh Rathi, Lauren J. O’Donnell

**Affiliations:** 1 School of Information and Communication Engineering, University of Electronic Science and Technology of China, Chengdu, China; 2 Brigham & Women’s Hospital and Harvard Medical School, Boston, Massachusetts, USA; 3 School of Computer Science and Engineering, The University of New South Wales, NSW, Australia; 4 School of Computer Science, The University of Sydney, NSW, Australia; 5 VA Boston Healthcare System and Harvard Medical School, Brockton, MA, USA; 6 Massachusetts General Hospital and Harvard Medical School, Boston, Massachusetts, USA

**Keywords:** Diffusion MRI, Tractography, White matter, Whole-brain analysis, Sex differences

## Abstract

Sex-specific characteristics demonstrate a substantial influence on the human brain white matter, suggesting distinct brain structural connectivity patterns between females and males. Diffusion MRI (dMRI) tractography is an important tool in assessing white matter connectivity and brain tissue microstructure across different populations. Whole brain white matter analysis using dMRI tractography for group statistical comparison is a challenging task due to the large number of white matter connections. In this work, we study whole-brain white matter connectivity differences between females and males using dMRI tractography. We study a large cohort of 707 healthy adult subjects from the Human Connectome Project Young Adult dataset. By applying a well-established fiber clustering pipeline and a suprathreshold fiber cluster statistical method, we evaluated tracts in the cerebral cortex, as well as those connecting to regions such as the cerebellum, which have been relatively less studied using dMRI tractography. We identified several tracts that differed significantly between females and males in terms of their fractional anisotropy and/or mean diffusivity. These included several deep white matter tracts (e.g., arcuate fasciculus, corticospinal tract, and corpus callosum) that have been previously shown to have sex differences, as well as superficial white matter tracts in the frontal lobe. However, there were relatively few cortical association tracts that exhibited significant sex differences. We also identified cerebellar tracts with sex differences. Finally, correlation analysis revealed that these white matter differences were linked to a range of neurobehavioral measures, with the strongest and most consistent associations observed for motor function. Overall, these findings provide characterizations of sex differences in the white matter and indicate that the circuits underlying motor function may be an important focus of future work on sex differences in the human brain.

## 1. Introduction

Sex-specific characteristics demonstrate a substantial influence on the human brain (Baron-Cohen et al., 2005; Cahill, 2006; Halpern, 2000). There has been an enduring interest in investigating neuroanatomical evidence of sexual dimorphism and its relationship to sex-specific behaviors (Giedd et al., 2012; Peper et al., 2011; Ruigrok et al., 2014). In particular, there is an evident prevalence of sex-specific differences in the brain white matter, suggesting distinct brain structural connectivity patterns between females and males (de Vries & Södersten, 2009; G. Gong et al., 2011). Diffusion magnetic resonance imaging (dMRI) (Basser et al., 1994) is the only existing non-invasive technique to map the brain white matter connections. dMRI measures the random molecular motion or diffusion of water molecules, allowing estimation of white matter fiber tracts in the brain via a process called tractography (Basser et al., 2000) and quantification of the microstructure of the white matter connections (Basser, 1995). Many studies have investigated white matter sexual dimorphism using dMRI tractography (Choi et al., 2010; Clayden et al., 2012; Eluvathingal et al., 2007; Hasan et al., 2009; Kitamura et al., 2011; Lebel et al., 2010; Lei et al., 2016; F. Liu et al., 2010; Y. Liu et al., 2011; Nordahl et al., 2015; Oh et al., 2007; Ritchie et al., 2018; Thiebaut de Schotten et al., 2011; G. Wang et al., 2014; J. Zhang et al., 2014; Daianu et al., 2012; Dennis et al., 2013; G. Gong et al., 2009; Herlin et al., 2024; Ingalhalikar et al., 2014; Jahanshad et al., 2011; Tyan et al., 2017; Wheelock et al., 2021; Yan et al., 2011). Among these, most existing studies have applied the traditional tract of interest analysis to investigate differences in specific anatomical tracts without considering the entire white matter (Choi et al., 2010; Clayden et al., 2012; Eluvathingal et al., 2007; Hasan et al., 2009; Herlin et al., 2024; Kitamura et al., 2011; Lebel et al., 2010; Lei et al., 2016; F. Liu et al., 2010; Y. Liu et al., 2011; Nordahl et al., 2015; Oh et al., 2007; Ritchie et al., 2018; Thiebaut de Schotten et al., 2011; G. Wang et al., 2014; Wheelock et al., 2021; J. Zhang et al., 2014). On the other hand, there is an increased interest in whole brain tractography analysis to assess sex-specific brain structural connectivity patterns across the entire brain (Daianu et al., 2012; Dennis et al., 2013; G. Gong et al., 2009, 2011; Ingalhalikar et al., 2014; Jahanshad et al., 2011; Tyan et al., 2017; Yan et al., 2011). In the present work, we propose to study sex-specific differences in the whole brain white matter connections using dMRI tractography.

Whole brain tractography analysis requires a parcellation of the tractography data to enable simultaneous analysis across a large number of white matter connections in the entire brain (F. Zhang et al., 2022). Performing whole brain tractography on one individual subject can generate hundreds of thousands of streamlines, which are not immediately useful for quantitative statistical analysis. Tractography parcellation is thus needed to divide the massive number of tractography streamlines into anatomically meaningful fiber tracts (bundles or fascicles). Currently, whole brain tractography studies on sex differences have focused on the traditional connectome-based analysis, which parcellates tractography based on white matter connections between brain cortical regions, enabling the study of the brain using graph theory (Daianu et al., 2012; Dennis et al., 2013; G. Gong et al., 2009; Ingalhalikar et al., 2014; Jahanshad et al., 2011; Tyan et al., 2017; Yan et al., 2011). Nevertheless, there are several known challenges in the application of connectome-style analyses to dMRI (Sotiropoulos & Zalesky, 2019), including anatomical variability of white matter tract terminations and cortical anatomy (Bajada et al., 2017; Hau et al., 2016), spatial distortions (Jones & Cercignani, 2010), false positive tracking (Maier-Hein et al., 2017), and reduced reproducibility due to the non-continuous nature of the matrix representation (Moyer et al., 2017).

Another popular method to perform whole brain tractography analysis applies the fiber clustering strategy (O’Donnell et al., 2013), which parcellates tractography by grouping the streamlines according to their geometric trajectories. Unlike traditional connectome analysis, which is gray-matter-centric and studies graph properties of the connectivity matrix between cortical regions, fiber clustering analysis is white-matter-centric and enables the study of the microstructural characteristics of brain connections with a focus on white matter anatomy. Fiber clustering has been shown to have several advantages compared to the cortical-parcellation-based method, including highly consistent tract identification across populations (Cetin-Karayumak et al., 2024; Ge et al., 2012; Li et al., 2024; F. Zhang et al., 2017; F. Zhang, Wu, Norton, et al., 2018; Ziyan et al., 2009), highly reproducible tractography parcellation (Kai & Khan, 2019; F. Zhang et al., 2019), robustness to the presence of false positive streamlines (Jin et al., 2014; Sydnor et al., 2018), and improved power for predicting human traits (R. Liu et al., 2023). Fiber clustering has been widely used to investigate the brain’s structural connectivity in various applications in health and disease (Fan et al., 2019; Lo et al., 2025; O’Donnell et al., 2017; Stojanovski et al., 2019; Tunç et al., 2016; Wu et al., 2018; F. Zhang, Savadjiev, et al., 2018), providing a useful tool to investigate white matter sex differences.

In this work, we propose to investigate sex differences in the whole brain white matter using dMRI tractography fiber clustering. We leverage a well-established data-driven fiber clustering white matter parcellation pipeline (Li et al., 2024; O’Donnell et al., 2012; O’Donnell & Westin, 2007; F. Zhang, Wu, Norton, et al., 2018), which has been successfully applied in multiple neuroscientific studies including brain disorder analysis (Irimia et al., 2020; Levitt et al., 2023; Wu et al., 2018; F. Zhang, Savadjiev, et al., 2018; F. Zhang, Wu, Ning, et al., 2018), neurosurgical planning research (S. Gong et al., 2018; O’Donnell et al., 2017; F. Zhang, Noh, et al., 2020), and brain atlasing (F. Zhang, Wu, Norton, et al., 2018; F. Zhang, Xie, et al., 2020). One of the advantages of the fiber clustering method is that, together with our white matter atlas (F. Zhang, Wu, Norton, et al., 2018), it enables a fine-grained whole brain tractography parcellation into a total of 1516 clusters. These clusters include not only fiber connections in the deep white matter but also superficial and cerebellar white matter connections that have been relatively less studied in previous work about sex differences. In addition, we utilize an advanced suprathreshold fiber cluster (STFC) statistical analysis method (F. Zhang, Wu, Ning, et al., 2018), specifically designed for the fiber clustering pipeline, for testing group differences in whole brain tractography. STFC enables simultaneous statistical group comparison across a large number of white matter connections. It leverages permutation testing and whole brain white matter geometry to enhance the statistical analysis of tractography while correcting for multiple comparisons. The STFC method has been successfully applied to study group differences in attention-deficit/hyperactivity disorder (ADHD) and healthy controls (F. Zhang, Wu, Ning, et al., 2018), showing a highly sensitive ability to detect white matter group differences.

We perform the study on a large healthy adult cohort (n = 707) from the Human Connectome Project Young Adult (HCP-YA) (Van Essen et al., 2013). The HCP-YA acquired high-quality *in vivo* macroscopic-level imaging data in an effort to elucidate the neural pathways and networks that underlie brain function and behavior (Barch et al., 2013). Non-imaging demographics (age, sex, etc.) and behavioral (fluid intelligence, language performance, etc.) measures are also available. Many research studies have leveraged the HCP-YA data and revealed important findings related to the human brain (Liégeois et al., 2019; Smith et al., 2015), including sex differences in brain gray matter and white matter volumes using T1-weighted MRI (Szalkai & Grolmusz, 2018) and in brain functional connectivity using functional MRI (Sen & Parhi, 2019; Weis et al., 2019; C. Zhang et al., 2018). A recent study utilized dMRI data to examine sex differences in major deep white matter fiber tracts using the HCP-YA cohort (Herlin et al., 2024). In our study, we aim to build upon this work by analyzing white matter connections across the entire brain, including the deep and superficial white matter, and further exploring their relationship to behavioral measures.

In the rest of this paper, we first describe the datasets under study, including dMRI data from a large cohort of 707 healthy adult subjects from the HCP-YA (Van Essen et al., 2013). Then, we introduce the related computational processes, including multi-tensor tractography and fiber clustering white matter parcellation, followed by the extraction of diffusion features of interest (fractional anisotropy (FA) and mean diffusivity (MD)). Next, we demonstrate the process of STFC statistical comparison to identify the white matter fiber clusters that are significantly different between the female and male groups. Following that, a correlation analysis is performed to investigate the relationship between the diffusion measures of the significant fiber clusters and the behavioral measures for each of the female and male groups. Finally, we give the experimental results and related discussion.

## 2. Methods

### 2.1. Datasets

We included a large cohort of 707 subjects (340 females and 367 males) in our study, with the following subject selection procedures from the entire HCP-YA population. (1) We excluded the 100 subjects that were used to generate the fiber clustering white matter atlas (F. Zhang, Wu, Ning, et al., 2018) (see Section 2.3 for a brief introduction of the atlas). This exclusion was to avoid any potential bias in the tractography parcellation across the subjects under study. (2) We excluded one of a twin pair if both twins participated in the HCP-YA study. (3) We selected the maximal subset of subjects that had a statistically similar age distribution between the male and female groups. In total, we selected 707 subjects, including 340 females and 367 males (mean age: 28.2±3.1y versus 28.0±3.6y, p=0.43).

The HCP-YA dMRI data was acquired with a high-quality image acquisition protocol using a customized Connectome Siemens scanner (Van Essen et al., 2012) and was processed following the well-designed HCP-YA minimum processing pipeline (Glasser et al., 2013). The acquisition parameters for the dMRI data were TE = 89.5 ms, TR = 5520 ms, phase partial Fourier = 6/8, voxel size = 1.25 × 1.25 × 1.25 mm^3^, 18 baseline images with a low diffusion weighting b = 5 s/mm^2^, and 270 diffusion-weighted images evenly distributed at three shells of b = 1000/2000/3000 s/mm^2^. More detailed information about the HCP-YA data acquisition and preprocessing can be found in (Glasser et al., 2013).

### 2.2. Multi-tensor whole brain tractography

Whole brain tractography was computed for each subject under study using the two-tensor Unscented Kalman Filter (UKF) method (Malcolm et al., 2010; Reddy & Rathi, 2016), as implemented in the *ukftractography* package (https://github.com/pnlbwh/ukftractography). The UKF method fits a mixture model of two tensors to the dMRI data while tracking, employing prior information from the previous step to help stabilize model fitting. The mixture model assumes there are two distinct tract directions within voxels, in which the first one represents the principal direction and the other one is from the second tract. This multi-tensor approach enables streamline-specific microstructure estimation. We chose the UKF method because it has been shown to be highly consistent in tracking streamlines in dMRI data from independently acquired populations across ages, health conditions, and image acquisitions (F. Zhang, Wu, Ning, et al., 2018), and it is more sensitive than standard single-tensor tractography, in particular in the presence of crossing fibers and peritumoral edema (Baumgartner et al., 2012; Chen et al., 2015, 2016; Liao et al., 2017). In addition, we chose the UKF method because it was used to generate tractography data in the fiber clustering atlas (F. Zhang, Wu, Ning, et al., 2018) (see Section 2.3 for a brief introduction of the atlas). Using the same tractography method can ensure highly consistent tract parcellation results across subjects (F. Zhang et al., 2017; F. Zhang, Wu, Ning, et al., 2018). We adopted the same tractography parameters as used in (F. Zhang, Wu, Ning, et al., 2018), producing about 1 million streamlines per subject. Visual and quantitative quality control of the tractography was performed using a quality control tool in the *whitematteranalysis* (WMA) software (https://github.com/SlicerDMRI/whitematteranalysis).

### 2.3. Tractography fiber clustering

Fiber clustering was performed to parcellate the whole brain tractography data for each individual subject using a well-established fiber clustering pipeline (O’Donnell et al., 2012; O’Donnell & Westin, 2007) and a fiber clustering atlas provided by the O’Donnell Research Group (ORG) (F. Zhang, Wu, Norton, et al., 2018). The ORG atlas contains an 800-cluster parcellation of the entire white matter and an anatomical fiber tract parcellation (http://dmri.slicer.org/atlases)^1^. The atlas was generated by creating dense tractography maps (using the same UKF tractography method as in the current study) of 100 individual HCP-YA subjects and then applying a fiber clustering method to group the tracts across subjects according to their similarity in shape and location. The resulting clusters were annotated using expert neuroanatomical knowledge. In the present study, we used the 800-cluster parcellation for whole brain white matter connectivity analysis, while the anatomical fiber tract parcellation was used to evaluate to which anatomical structure the identified significantly different white matter connections belonged.

The fiber clustering method was applied to perform white matter parcellation of one subject as follows. A tractography-based registration was performed to align the subject’s tractography data into the atlas space. A fiber spectral embedding was conducted to compute the similarity of streamlines between the subject and the atlas, followed by the assignment of each streamline of the subject to the corresponding atlas cluster. This process produced a whole brain white matter parcellation into 800 clusters. These clusters included 84 commissural clusters as well as 716 bilateral hemispheric clusters (that included streamlines in both hemispheres). We separated the hemispheric clusters by hemisphere, thus resulting in (716 × 2 + 84) = 1516 clusters per subject. All fiber clustering processing was performed using the *whitematteranalysis* software (https://github.com/SlicerDMRI/whitematteranalysis), and all parameters were set to their default values. (Details are described in (O’Donnell et al., 2012, 2017; O’Donnell & Westin, 2007; F. Zhang, Wu, Norton, et al., 2018).)

### 2.4. Quantitative diffusion measure computation

Diffusion measures were extracted from each fiber parcel to quantify the microstructural properties of the brain white matter connections. In this study, we focused on the FA and MD measures. FA and MD are quantitative microstructure measures derived from modeling water diffusion using dMRI mathematics (Basser, 1995). They are sensitive to the differences in the underlying cellular microstructure in brain tissues. Studies in the literature have observed differences in FA and/or MD in multiple white matter connections between females and males (Choi et al., 2010; Eluvathingal et al., 2007; Kitamura et al., 2011; Lebel et al., 2010; F. Liu et al., 2010; Y. Liu et al., 2011; Oh et al., 2007; Thiebaut de Schotten et al., 2011). Specifically, in the present study, we computed the mean FA and the mean MD of each fiber parcel.

### 2.5. STFC statistical comparison for group differences

For each diffusion measure, statistical group comparison was performed using the STFC method (F. Zhang, Wu, Ning, et al., 2018). The STFC method leverages permutation testing and whole brain white matter geometry to enhance the statistical analysis of tractography. The basic idea of the STFC method is similar to the well-known voxel-cluster-thresholding approach (Nichols & Holmes, 2002; Smith & Nichols, 2009), but with the improvement of leveraging a fiber parcel’s neighborhood (nearby parcels with similar white matter anatomy) to support each parcel’s statistical significance when correcting for multiple comparisons.

The STFC method employs several steps, as described in detail in the original publication (F. Zhang, Wu, Ning, et al., 2018). Here, we give a brief overview of the steps of the STFC method as employed in this study. Specifically, for each diffusion measure (FA or MD), the STFC method first analyzed each fiber cluster using a general linear model (GLM) to compare between the female and male groups, with age as a covariate to control for potential age differences. Next, fiber clusters with uncorrected p<0.05 from the GLM analysis were identified. Then, these identified fiber clusters were combined with their neighbors to form STFCs, which contained multiple neighboring clusters, where neighborhoods were defined based on streamline geometric similarity. Finally, a nonparametric permutation test was used to determine a corrected p-value for each STFC based on its STFC size, i.e., the number of fiber clusters within the STFC, under the null hypothesis of no group difference in any cluster. The STFCs with a corrected p<0.05 were considered to be significantly different between the female and male groups.

### 2.6. Canonical correlation analysis of behavioral measures

We then investigated the relationship between the diffusion measures of the identified STFCs and the behavioral measures for each of the female and male groups. We adopted the sophisticated multivariate analysis method proposed in (Smith et al., 2015), which was designed to study the relationship between functional MRI connectomes and behavioral data in HCP. This method uses canonical correlation analysis (CCA) (Hotelling, 1936) to identify pairs of canonical variates along which sets of behavioral measures and patterns of imaging measures co-vary in a similar way across subjects. Each pair of variates is referred to as a mode of co-variation, associated with a coefficient *r* that indicates the strength of the canonical correlation.

We investigated the behavioral measures belonging to the cognition, emotion, motor, personality, and sensory categories, respectively, provided in the HCP-YA database (as documented in HCP-YA S1200 Data Dictionary). For each diffusion measure (FA or MD), we performed two CCA analyses to assess relationships between the behavioral measures and the diffusion measures of the significant STFCs in the female and male groups, respectively. Thus, for each behavioral category, there were a total of 4 CCA analyses across the two diffusion measures and two groups. For each CCA analysis, the major mode (i.e., the one with the highest coefficient *r*) was reported. A permutation test was performed to determine the statistical significance of the major mode by testing for a null distribution of *r* via 100,000 permutations of the behavioral measures relative to the diffusion measures (Smith et al., 2015). A p-value lower than 0.05 (FDR corrected for multiple comparisons across the four modes) was considered statistically significant.

## 3. Results

### 3.1. Sex differences in white matter FA values

**Figure 1.**
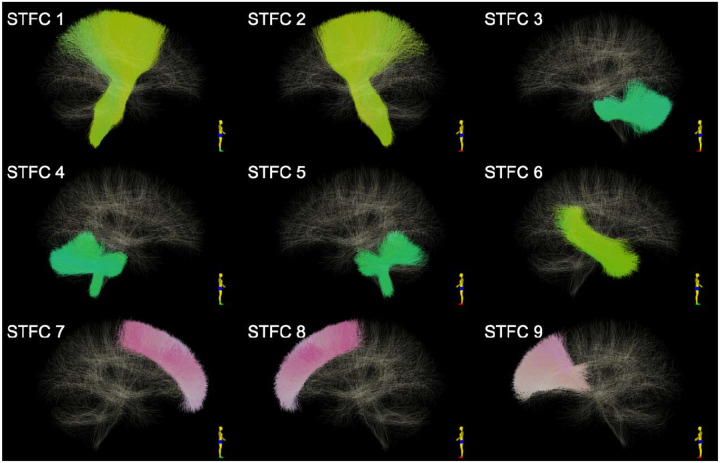
Visualization of the STFCs that are identified to be significantly different between females and males using FA.

The STFC identified nine white matter regions that exhibited significant sex differences in fractional anisotropy Table 1; Figure 1). These differences were observed in several white matter pathways that showed a significantly larger FA in females: coronal radiata bilaterally, middle and inferior cerebellar peduncles, and right thalamo-temporal projections. Males exhibited a larger FA in the superficial frontal white matter bilaterally and in the left thalamo-frontal projection.

### 3.2. Sex differences in white matter MD values

**Figure 2.**
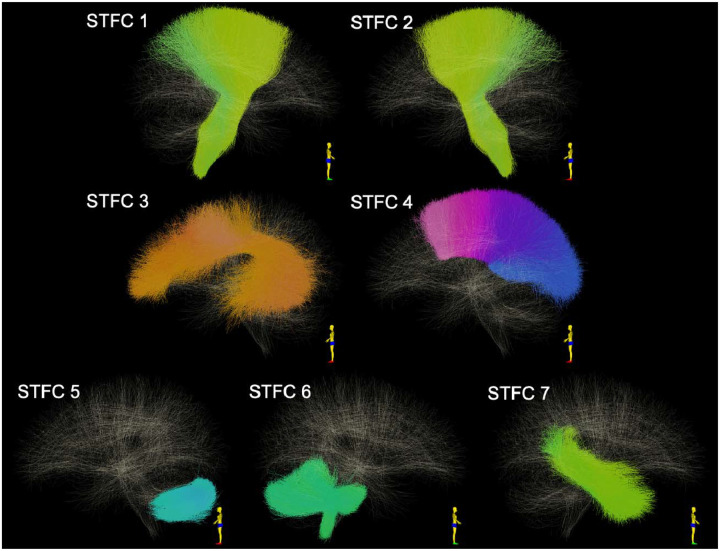
Visualization of the STFCs that are identified as significantly different between females and males using MD.

The STFC approach identified seven white matter regions that exhibited significant sex differences in mean diffusivity (Table 2; Figure 2). Females exhibited lower MD in the coronal radiata bilaterally, the left arcuate fascicle, and the corpus callosum (subdivisions 4 to 7). By contrast, males had lower MD in the left intracerebellar parallel tract, the right middle/inferior cerebellar peduncle, and the right thalamo-temporal projection.

### 3.3. Canonical correlation with behavioral metrics

Canonical correlation analyses were performed to investigate the relationship between the white matter microstructure and behavior in females and males. Analysis was performed separately for FA and MD, and five behavioral domains (cognition, emotion, motor, personality, sensory) from the HCP-YA dataset were evaluated (Table 3). In the female group, FA values in the significant STFC were correlated with motor performance and sensory processing, while in males, FA was correlated only with motor performance. For MD, significant correlations were identified in the female group with cognitive performance and motor behavior, whereas in males, MD was again significantly correlated with motor performance.

## 4. Discussion

In this study, we investigated sex differences in the whole brain white matter using dMRI tractography fiber clustering. We performed whole brain fiber tracking using a multi-fiber UKF tractography method (Malcolm et al., 2010; Reddy & Rathi, 2016). We leveraged a well-established data-driven fiber clustering pipeline (O’Donnell et al., 2012; O’Donnell & Westin, 2007) and an anatomically curated white matter atlas (F. Zhang, Wu, Norton, et al., 2018) for tractography parcellation. We extracted two diffusion measures of interest (FA and MD) for the quantification of the tissue microstructure of each fiber cluster. We adopted the advanced STFC method for statistical between-group comparison (F. Zhang, Wu, Ning, et al., 2018). A large population, including a total of 707 healthy adult subjects (340 females and 367 males) from the HCP-YA database, was studied. Overall, we found sex differences in the whole brain white matter connectivity of the human brain.

We have several specific observations regarding the comparison results between the female and male groups under study. First, we found multiple significantly different white matter connections from the entire brain (a total of 16 STFCs). These white matter connections included several deep white matter tracts (e.g., arcuate fasciculus, corticospinal tract, and corpus callosum) that have been previously shown to have sex differences (see below for related work and detailed discussion). We also identified fiber tracts with significant sex differences from the superficial and the cerebellar white matter regions that have been relatively less investigated before using dMRI tractography. Second, there was no consensus on the direction of differences in FA and MD across the multiple identified STFCs. Nine STFCs were identified using FA, of which 4 had significantly higher FA in the female group and 3 had significantly higher FA in the male group. Six STFCs were identified using MD, of which 4 had significantly higher MD in the female group and 2 had significantly higher MD in the male group. Third, with the CCA analysis between the tract microstructure measures and the behavioral data, we found that the identified white matter connections were related to multiple brain functions, including motor, sensory, and cognitive functions. Significant correlations with motor function were present in both groups, while identified connections showed significant correlations with cognition and sensory processing in the female group and sensory processing in the male group.

Numerous studies have applied dMRI to study sex-specific differences in the brain’s white matter. The first category of studies has focused on voxel-based analysis. Traditional voxel-based dMRI studies have been conducted using region-of-interest (ROI) methods to identify white matter differences in a local region (Szeszko et al., 2003). Other voxel-based studies have performed whole brain white matter analysis using approaches such as voxel-based morphometry (VBM) (Dunst et al., 2014) and tract-based spatial statistics (TBSS) (Bava et al., 2011; Y. Wang et al., 2012). These voxel-based studies have revealed substantial sex-specific neuroanatomical characteristics in the white matter. However, using voxel-based methods, accurate localization of between-population differences to specific white matter tracts can be difficult to assess (Bach et al., 2014; Davatzikos, 2004), especially since more than one tract may cross within a single voxel (Raffelt et al., 2015; F. Zhang, Wu, Ning, et al., 2018).

Another category of dMRI studies has applied tractography-based analysis to investigate white matter sex differences. Tractography allows estimation of the trajectories of fiber tracts (Pujol et al., 2015; Yamada et al., 2009) and thus measurement of microstructural white matter properties of fiber pathways for a more detailed investigation of specific subpopulations of fibers (Ciccarelli et al., 2008; Hagmann et al., 2007). Hypothesis-driven tractography studies using tract-of-interest approaches have identified sex differences in many individual white matter fiber tracts (Choi et al., 2010; Clayden et al., 2012; Eluvathingal et al., 2007; Hasan et al., 2009; Herlin et al., 2024; Kitamura et al., 2011; Lebel et al., 2010; Lei et al., 2016; F. Liu et al., 2010; Y. Liu et al., 2011; Nordahl et al., 2015; Oh et al., 2007; Ritchie et al., 2018; Thiebaut de Schotten et al., 2011; G. Wang et al., 2014; J. Zhang et al., 2014). Despite differences in methodology, our study’s findings are in agreement with several recent tract-based findings in the HCP-YA dataset, including sex differences of microstructural parameters in the corticospinal tract (part of the corona radiata region we identified), cerebellar connections, and thalamo-temporal radiations (Herlin et al., 2024). Another category of tractography-based studies, to which our work belongs, has performed whole brain white matter analysis (Daianu et al., 2012; Dennis et al., 2013; G. Gong et al., 2009, 2011; Ingalhalikar et al., 2014; Jahanshad et al., 2011; Tyan et al., 2017; Yan et al., 2011). The benefits of whole brain tractography analysis include the assessment of sex-specific brain structural connectivity patterns across the entire brain, instead of specific tracts of interest. Recent neuroimaging studies, including not only dMRI but also other imaging modalities such as structural T1- and T2-weighted MRI, functional MRI, and positron emission tomography (PET), have shown sex-related differences likely exist in the whole brain (see a review (G. Gong et al., 2011) and several more recent studies (Hu et al., 2013; Tomasi & Volkow, 2012; Xin et al., 2019)). Another benefit of whole brain tractography analysis is enabling the data-driven identification of fiber tracts that have group differences (F. Zhang, Savadjiev, et al., 2018).

In the related literature of whole brain tractography sex difference studies, the methodology of cortical-parcellation-based connectome analysis has been widely used. In connectome analysis for studies of sex differences, a matrix of connection strengths between cortical regions is created, enabling the study of the brain using graph theory (Daianu et al., 2012; Dennis et al., 2013; G. Gong et al., 2009; Ingalhalikar et al., 2014; Jahanshad et al., 2011; Tyan et al., 2017; Yan et al., 2011). For instance, Ingalhalikar et al. constructed a connectome matrix based on a cortical/subcortical parcellation including 95 regions of interest (ROIs) and the number of tractography streamlines between each pair of the ROIs (thus leading to a 95 × 95 matrix). Multiple connectome measures have been studied by comparing graph theoretical properties across sexes (e.g., modularity, a measure of how well the network can be delineated into groups (see (Ingalhalikar et al., 2014) for details). Other widely studied connectome measures include small-worldness, weighted network cost, and clustering coefficient (G. Gong et al., 2009; Tyan et al., 2017). While connectome-style analyses have been widely used to study sex-specific brain connectivity differences, there are several technical challenges, including anatomical variability of fiber tract terminations and cortical anatomy (Bajada et al., 2017; Hau et al., 2016), spatial distortions (Jones & Cercignani, 2010), and reduced reproducibility due to the non-continuous nature of the matrix representation (Moyer et al., 2017). In our work, we applied another whole brain white matter connectivity analysis, i.e., dMRI tractography fiber clustering. In contrast, fiber cluster analysis is white-matter-centric, leveraging information about the full course of a fiber tract, which is the anatomical basis for fiber tract definition (O’Donnell et al., 2013; Wassermann et al., 2016). Previous studies have indicated that white-matter-centric approaches benefit from low variability across subjects in terms of the white matter parcellations that can be defined (Chekir et al., 2017; Guevara et al., 2017; O’Donnell et al., 2017; F. Zhang et al., 2017), and highly consistent tract identification across populations (Ge et al., 2012; Sydnor et al., 2018; F. Zhang et al., 2017, 2019; F. Zhang, Wu, Norton, et al., 2018; Ziyan et al., 2009), and highly reliable and reproducible performance for white matter parcellation (F. Zhang et al., 2019).

Whole brain analysis is intended to identify which connections are more different, which requires enhanced statistical power. In our study, we used a data-driven whole brain white matter atlas that finely divides all input tractography into many regions (including a total of 800 fiber clusters) and hence allows the identification of potential local white matter group differences from the whole brain. In addition, we applied our suprathreshold fiber cluster (STFC) method that leverages the whole brain fiber geometry to enhance statistical group difference analyses (F. Zhang, Wu, Ning, et al., 2018). The method performs statistical analyses of tractography for group comparison while correcting for multiple comparisons to allow simultaneous analysis across the entire white matter.

The identified white matter tracts that had significant sex differences in our study were obtained using the multi-fiber UKF tractography. The UKF has been shown to be more sensitive in tracking through the regions in the presence of crossing fibers (S. Gong et al., 2018; He et al., 2023; Liao et al., 2017; Xie et al., 2020; F. Zhang, Wu, Norton, et al., 2018). This could reduce the well-known false negative tractography problem and enable the investigation of more white matter connections (e.g., the corticospinal tract and arcuate fasciculus) compared to traditional single-tensor DTI tractography (Duffau, 2014; Jeurissen et al., 2013; Nimsky, 2014). A few sex difference studies have applied multi-tensor/fiber tractography for tract-ofinterest analysis (Benavidez et al., 2024; Clayden et al., 2012; Kang et al., 2025; Y. Liu et al., 2011; Ritchie et al., 2018).

Next, we discuss detailed observations regarding the results for each significantly different white matter connection. Multiple identified deep white matter fiber tracts correspond to white matter structures that have been previously implicated to have sex differences, while we have also identified several cerebellar tracts and superficial tracts that have been rarely reported in the existing studies.

Bilateral tracts from the corona radiata to the brain stem, including the CSTs, were significantly different between the female and male groups under study in both FA and MD. Multiple studies have reported sex-specific differences in these white matter regions (Bava et al., 2011; Hervé et al., 2009; Seunarine et al., 2016; Takao et al., 2014). The corona radiata and CST are known to be related to human sensory and motor functions, which potentially explain the sensory and motor correlations in our CCA analysis. It is interesting to note that both FA and MD were significantly higher in the female group, suggesting greater diffusion anisotropy and diffusivity compared to the male group. Bava et al have reported a similar finding in bilateral CSTs using a voxel-based TBSS analysis (Bava et al., 2011).

The posterior portion of the corpus callosum tract was found to have significant sex differences in MD but not in FA in our study. The corpus callosum is one of the most widely studied white matter structures in the literature of dMRI-based sex differences. Early studies have identified the differences in the size of the corpus callosum between sexes, in particular, that of the corpus callosum splenium (the posterior portion) (Bishop & Wahlsten, 1997; DeLacoste-Utamsing & Holloway, 1982). Multiple studies have also identified tissue microstructural differences in the corpus callosum using voxel-based dMRI analysis, mostly with the findings of lower diffusion anisotropy and/or higher mean diffusivity in females (Menzler et al., 2011; Shin et al., 2005; Westerhausen et al., 2003). Our results are in line with these studies by showing a higher MD in the female group; however, we did not find any differences in the corpus callosum using FA. Our identified corpus callosum tract was specifically from the CC4 to CC7 (Makris et al., 1999), connecting to the motor cortex (CC4), the somatosensory cortex (CC5), the superior parietal lobe (CC6), and the lateral occipital cortex (CC7).

We identified the left arcuate fasciculus to be significantly different, where the female group had a significantly higher MD. The left arcuate fasciculus is known to be related to human language function (Zekelman et al., 2022). Our results in the left arcuate fasciculus, but not in the contralateral side, are possibly related to the sex difference of left-hemispheric language lateralization that has been reported in many studies (Allendorfer et al., 2016; Catani et al., 2007). Several studies have reported sex differences in the left arcuate fasciculus, mostly using FA (Jung et al., 2019; Madhavan et al., 2014) (significantly lower FA in females). Nevertheless, in our study, no FA differences were identified in the arcuate fasciculus.

We found a significantly higher FA of the left striato-frontal tract in the male group. The striato-frontal structural connections are known to play important roles in behaviors such as reward-related processes and impulse control (Morris et al., 2016). Previous research has hinted at potential sex differences in the striato-frontal connections. For example, multiple voxel-wise studies have shown that males had stronger white matter connections in white matter regions and tracts that underlie striatal-frontal connections (Lei et al., 2016), which is in line with our finding that the male group has a higher FA. More recently, we and another group have also identified sex differences with a tractography technique by measuring topographical organization (F. Zhang, Vangel, et al., 2020) and tract volume (Lei et al., 2016) in striato-frontal white matter connections. While bilateral differences were identified in these two studies as well as in the aforementioned voxel-wise study (Lei et al., 2016), in the present study, we only identified significant differences in the left hemisphere.

We also found that multiple cerebellar white matter tracts were significantly different in both FA and MD. The cerebellum is important and related to multiple brain functions (Mormina et al., 2017; Travis et al., 2015). Compared to the deep white matter, sex differences of the cerebellar white matter are relatively less studied, in particular using dMRI. A few studies have identified microstructure differences between sexes in the cerebellar peduncle (Bede et al., 2014; Hosoki et al., 2023; Kanaan et al., 2012, 2014; Takao et al., 2014) using voxel-based dMRI analysis. One dMRI tractography study has identified sex differences in the structural connectome between the cerebellum and the cerebral cortex (Ingalhalikar et al., 2014). Our results provided additional evidence for sexual dimorphism in the cerebellar white matter. We showed that there were significant sex differences in the middle cerebellar peduncle tract, particularly in the right hemisphere where both FA and MD were significantly different. Another interesting finding is that we found intracerebellar parallel tract connections were significantly different in MD, which has not yet been reported. Intracerebellar parallel tract connections are important and shown to be linked with sensorimotor functions (Jörntell, 2017).

Bilateral superficial white matter tracts in the superior frontal lobes were found to be significantly different between the female and male groups. Unlike the deep white matter structures that have been extensively studied in different clinical and scientific applications, the superficial white matter is relatively less studied (Phillips et al., 2013; Shiino et al., 2017). In our study, we find that only FA showed sex differences in the superficial white matter, where the female group had lower FA.

An important aspect of the current study is that it was performed in sufficient numbers to assess not only the fiber tracts that are significantly different between males and females, but also those fiber bundles that did not exhibit sex differences. Only a few short and long-range cortico-cortical association bundles differed on the basis of sex, suggesting limited differences between females and males. The superficial white matter of the frontal lobe had a higher FA in males, whereas the left arcuate fasciculus and the corpus callosum exhibited higher MD in females. FA and MD in the short association cortico-cortical white matter systems, such as the superficial white matter in the temporal, occipital, and parietal areas, did not vary according to sex., Moreover, most long-range cortico-cortical fiber bundles did not differ with sex (i.e., cingulum bundle, external capsule, extreme capsule, ILF, iFOF, MDLF, SLF, UF), suggesting that sex differences in cortical processing are limited.

Examination of cerebellar tracts revealed considerable sex differences, with the middle cerebellar peduncles being consistently affected. When taken together with the findings from the cerebral cortex, the most consistent findings may be related to motor function. Motor function and planning are largely localized to the frontal lobe, and bilateral differences in the frontal lobe superficial white matter, and the corticospinal and corticopontine tracts pass through the coronal radiata. In addition, the middle cerebellar peduncle, which carries signals from the corticopontine tract after relay in the ipsilateral pontine nucleus, relays motor signals to the cerebellum. These findings are consistent with the correlation analyses that show the significantly different tracts are most consistently related to neurobehavioral indices of motor function.

Our findings are broadly consistent with investigations using dMRI tractography in adolescents, which have shown males to have a larger within hemispheric connectivity, but lower cerebellar connectivity (Ingalhalikar et al., 2014).

## 5. Conclusion

We studied whole-brain white matter sex differences using a data-driven fiber clustering pipeline, a white matter parcellation atlas, and an advanced STFC statistical analysis method. We compared FA and MD between the two groups in a large cohort of 707 subjects. We identified multiple fiber tracts that had significant differences, which provides evidence pointing to sex-specific brain white matter connectivity.

## Figures and Tables

**Table 1.** Significant differences in white matter regions between females and males using FA.

STFC index	STFC size	Anatomical structure	p-value	Mean FA of clusters in STFC	
				Female group (Mean±SD)	Male group (Mean±SD)
STFC 1	11	Corona radiata (right)	0.0105	**0.6431 ± 0.0208**	0.6350 ± 0.0218
STFC 2	13	Corona radiata (left)	0.0036	**0.6333 ± 0.0204**	0.6246 ± 0.0215
STFC 3	8	Middle cerebellar peduncle	0.0422	**0.5213 ± 0.0235**	0.5100 ± 0.0272
STFC 4	13	Middle/inferior cerebellar peduncle (right)	0.0036	**0.4021 ± 0.0234**	0.3856 ± 0.0303
STFC 5	8	Middle/inferior cerebellar peduncle (left)	0.0422	**0.4004 ± 0.0256**	0.3773 ± 0.0308
STFC 6	8	Thalamo-temporal (right)	0.0422	**0.3695 ± 0.0222**	0.3614 ± 0.0243
STFC 7	15	Superficial frontal (right)	0.0012	0.3894 ± 0.0269	**0.4011 ± 0.0259**
STFC 8	15	Superficial frontal (left)	0.0012	0.3849 ± 0.0261	**0.3970 ± 0.0266**
STFC 9	12	Thalamo-frontal (left)	0.0062	0.3821 ± 0.0245	**0.3976 ± 0.0268**

**Table 2.** Significant differences in white matter regions between females and males using MD.

STFC index	STFC size	Anatomical structure	p-value	Mean MD of clusters in STFC (10^−3^)	
				Female group (Mean±SD)	Male group (Mean±SD)
STFC 1	12	Corona radiata (left)	0.0263	**0.1303 ± 0.0027**	0.1288 ± 0.0027
STFC 2	12	Corona radiata (right)	0.0263	**0.1297 ± 0.0026**	0.1280 ± 0.0027
STFC 3	12	Arcuate fasciculus (left)	0.0263	**0.1276 ± 0.0034**	0.1258 ± 0.0035
STFC 4	18	Corpus callosum (4–7)	0.0002	**0.1179 ± 0.0030**	0.1159 ± 0.0029
STFC 5	12	Intracerebellar parallel tract (left)	0.0263	0.1371 ± 0.0037	**0.1389 ± 0.0044**
STFC 6	12	Middle/inferior cerebellar peduncle (right)	0.0263	0.1496 ± 0.0035	**0.1513 ± 0.0037**
STFC 7	20	Thalamo-temporal (right)	0.0001	0.1329 ± 0.0036	**0.1358 ± 0.0042**

**Table 3:** CCA analysis between STFC diffusion measures and subject behavioral measures. Correlation coefficient and p-value (permutation testing, with FDR correction) are reported.

		Cognition	Emotion	Motor	Personality	Sensory
FA	Female	0.421 (p=0.082)	0.400 (p=0.361)	0.321 (**p=0.015**)	0.268 (p=0.259)	0.412 (**p=0.002**)
	Male	0.395 (p=0.157)	0.378 (p=0.433)	0.301 (**p=0.016**)	0.256 (p=0.259)	0.301 (p=0.259)
MD	Female	0.429 (**p=0.021**)	0.416 (p=0.101)	0.289 (**p=0.025**)	0.187 (p=0.867)	0.290 (p=0.237)
	Male	0.363 (p=0.237)	0.360 (p=0.469)	0.304 (**p=0.006**)	0.241 (p=0.237)	0.300 (p=0.136)

## Data Availability

The data under study are from the public Human Connectome Project (HCP) dataset (www.humanconnectome.org). The UKF code to perform fiber tracking is publicly available (https://github.com/pnlbwh/ukftractography). The WMA code to perform fiber clustering is publicly available (https://github.com/SlicerDMRI/whitematteranalysis). The STFC code to perform the statistical analysis is publicly available (https://github.com/zhangfanmark/STFC). Tract visualization is performed using SlicerDMRI (https://dmri.slicer.org).

## Footnotes

1 The anatomical tract parcellation includes 58 deep white matter fiber tracts, plus 198 short and medium range superficial fiber clusters organized into 16 categories according to the brain lobes they connect.

## References

[R1] Allendorfer J. B., Hernando K. A., Hossain S., Nenert R., Holland S. K., & Szaflarski J. P. (2016). Arcuate fasciculus asymmetry has a hand in language function but not handedness. Human Brain Mapping, 37(9), 3297–3309. 27144738 10.1002/hbm.23241 PMC4988400

[R2] Bach M., Laun F. B., Leemans A., Tax C. M. W., Biessels G. J., Stieltjes B., & Maier-Hein K. H. (2014). Methodological considerations on tract-based spatial statistics (TBSS). NeuroImage, 100, 358–369. 24945661 10.1016/j.neuroimage.2014.06.021

[R3] Bajada C. J., Haroon H. A., Azadbakht H., Parker G. J. M., Lambon Ralph M. A., & Cloutman L. L. (2017). The tract terminations in the temporal lobe: Their location and associated functions. Cortex; a Journal Devoted to the Study of the Nervous System and Behavior, 97, 277–290. 27118049 10.1016/j.cortex.2016.03.013 PMC5726606

[R4] Barch D. M., Burgess G. C., Harms M. P., Petersen S. E., Schlaggar B. L., Corbetta M., Glasser M. F., Curtiss S., Dixit S., Feldt C., Nolan D., Bryant E., Hartley T., Footer O., Bjork J. M., Poldrack R., Smith S., Johansen-Berg H., Snyder A. Z., … WU-Minn HCP Consortium. (2013). Function in the human connectome: task-fMRI and individual differences in behavior. NeuroImage, 80, 169–189. 23684877 10.1016/j.neuroimage.2013.05.033 PMC4011498

[R5] Baron-Cohen S., Knickmeyer R. C., & Belmonte M. K. (2005). Sex differences in the brain: implications for explaining autism. Science, 310(5749), 819–823. 16272115 10.1126/science.1115455

[R6] Basser P. J. (1995). Inferring microstructural features and the physiological state of tissues from diffusion-weighted images. NMR in Biomedicine, 8(7), 333–344. 8739270 10.1002/nbm.1940080707

[R7] Basser P. J., Mattiello J., & LeBihan D. (1994). MR diffusion tensor spectroscopy and imaging. Biophysical Journal, 66(1), 259–267. 8130344 10.1016/S0006-3495(94)80775-1 PMC1275686

[R8] Basser P. J., Pajevic S., Pierpaoli C., Duda J., & Aldroubi A. (2000). In vivo fiber tractography using DT-MRI data. Magnetic Resonance in Medicine: Official Journal of the Society of Magnetic Resonance in Medicine / Society of Magnetic Resonance in Medicine, 44(4), 625–632. 10.1002/1522-2594(200010)44:4<625::aid-mrm17>3.0.co;2-o 11025519

[R9] Baumgartner C., Michailovich O., Levitt J., Pasternak O., Bouix S., Westin C., & Rathi Y. (2012). A unified tractography framework for comparing diffusion models on clinical scans. Computational Diffusion MRI Workshop of MICCAI, 27–32.

[R10] Bava S., Boucquey V., Goldenberg D., Thayer R. E., Ward M., Jacobus J., & Tapert S. F. (2011). Sex differences in adolescent white matter architecture. Brain Research, 1375, 41–48. 21172320 10.1016/j.brainres.2010.12.051 PMC3035918

[R11] Bede P., Elamin M., Byrne S., & Hardiman O. (2014). Sexual dimorphism in ALS: exploring gender-specific neuroimaging signatures. Amyotrophic Lateral Sclerosis & Frontotemporal Degeneration, 15(3–4), 235–243. 24344910 10.3109/21678421.2013.865749

[R12] Benavidez S. M., Abaryan Z., Kim G. S., Laltoo E., McCracken J. T., Thompson P. M., & Lawrence K. E. (2024). Sex Differences in the Brain’s White Matter Microstructure during Development assessed using Advanced Diffusion MRI Models. 2024 46th Annual International Conference of the IEEE Engineering in Medicine and Biology Society (EMBC), 1–7. 10.1109/EMBC53108.2024.10781992 40039411

[R13] Bishop K. M., & Wahlsten D. (1997). Sex differences in the human corpus callosum: myth or reality? Neuroscience and Biobehavioral Reviews, 21(5), 581–601. 9353793 10.1016/s0149-7634(96)00049-8

[R14] Cahill L. (2006). Why sex matters for neuroscience. Nature Reviews. Neuroscience, 7(6), 477–484. 16688123 10.1038/nrn1909

[R15] Catani M., Allin M. P. G., Husain M., Pugliese L., Mesulam M. M., Murray R. M., & Jones D. K. (2007). Symmetries in human brain language pathways correlate with verbal recall. Proceedings of the National Academy of Sciences of the United States of America, 104(43), 17163–17168. 17939998 10.1073/pnas.0702116104 PMC2040413

[R16] Cetin-Karayumak S., Zhang F., Zurrin R., Billah T., Zekelman L., Makris N., Pieper S., O’Donnell L. J., & Rathi Y. (2024). Harmonized diffusion MRI data and white matter measures from the Adolescent Brain Cognitive Development Study. Scientific Data, 11(1), 249. 38413633 10.1038/s41597-024-03058-w PMC10899197

[R17] Chekir A., Hassas S., Descoteaux M., Côté M., Garyfallidis E., & Oulebsir-Boumghar F. (2017). 3D-SSF: A bio-inspired approach for dynamic multi-subject clustering of white matter tracts. Computers in Biology and Medicine, 83, 10–21. 28188985 10.1016/j.compbiomed.2017.01.016

[R18] Chen Z., Tie Y., Olubiyi O., Rigolo L., Mehrtash A., Norton I., Pasternak O., Rathi Y., Golby A. J., & O’Donnell L. J. (2015). Reconstruction of the arcuate fasciculus for surgical planning in the setting of peritumoral edema using two-tensor unscented Kalman filter tractography. NeuroImage. Clinical, 7, 815–822. 26082890 10.1016/j.nicl.2015.03.009 PMC4459040

[R19] Chen Z., Tie Y., Olubiyi O., Zhang F., Mehrtash A., Rigolo L., Kahali P., Norton I., Pasternak O., Rathi Y., Golby A. J., & O’Donnell L. J. (2016). Corticospinal tract modeling for neurosurgical planning by tracking through regions of peritumoral edema and crossing fibers using two-tensor unscented Kalman filter tractography. International Journal of Computer Assisted Radiology and Surgery, 11(8), 1475–1486. 26762104 10.1007/s11548-015-1344-5 PMC4942409

[R20] Choi C.-H., Lee J.-M., Koo B.-B., Park J. S., Kim D.-S., Kwon J. S., & Kim I. Y. (2010). Sex differences in the temporal lobe white matter and the corpus callosum: a diffusion tensor tractography study. Neuroreport, 21(1), 73–77. 19996809 10.1097/WNR.0b013e3283345eb0

[R21] Ciccarelli O., Catani M., Johansen-Berg H., Clark C., & Thompson A. (2008). Diffusion-based tractography in neurological disorders: concepts, applications, and future developments. Lancet Neurology, 7(8), 715–727. 18635020 10.1016/S1474-4422(08)70163-7

[R22] Clayden J. D., Jentschke S., Muñoz M., Cooper J. M., Chadwick M. J., Banks T., Clark C. A., & Vargha-Khadem F. (2012). Normative development of white matter tracts: similarities and differences in relation to age, gender, and intelligence. Cerebral Cortex, 22(8), 1738–1747. 21940703 10.1093/cercor/bhr243

[R23] Daianu M., Jahanshad N., Dennis E. L., Toga A. W., McMahon K. L., de Zubicaray G. I., Martin N. G., Wright M. J., Hickie I. B., & Thompson P. M. (2012). Left versus right hemisphere differences in brain connectivity: 4-Tesla HARDI tractography in 569 twins. 2012 9th IEEE International Symposium on Biomedical Imaging (ISBI), 2012, 526–529. 10.1109/ISBI.2012.6235601 PMC4232939 25404993

[R24] Davatzikos C. (2004). Why voxel-based morphometric analysis should be used with great caution when characterizing group differences. NeuroImage, 23(1), 17–20. 15325347 10.1016/j.neuroimage.2004.05.010

[R25] DeLacoste-Utamsing C., & Holloway R. L. (1982). Sexual dimorphism in the human corpus callosum. Science, 216(4553), 1431–1432. 7089533 10.1126/science.7089533

[R26] Dennis E. L., Jahanshad N., McMahon K. L., de Zubicaray G. I., Martin N. G., Hickie I. B., Toga A. W., Wright M. J., & Thompson P. M. (2013). Development of brain structural connectivity between ages 12 and 30: a 4-Tesla diffusion imaging study in 439 adolescents and adults. NeuroImage, 64, 671–684. 22982357 10.1016/j.neuroimage.2012.09.004 PMC3603574

[R27] de Vries G. J., & Södersten P. (2009). Sex differences in the brain: the relation between structure and function. Hormones and Behavior, 55(5), 589–596. 19446075 10.1016/j.yhbeh.2009.03.012 PMC3932614

[R28] Duffau H. (2014). Diffusion tensor imaging is a research and educational tool, but not yet a clinical tool. World Neurosurgery, 82(1–2), e43–e45. 24017954 10.1016/j.wneu.2013.08.054

[R29] Dunst B., Benedek M., Koschutnig K., Jauk E., & Neubauer A. C. (2014). Sex differences in the IQ-white matter microstructure relationship: a DTI study. Brain and Cognition, 91, 71–78. 25238623 10.1016/j.bandc.2014.08.006 PMC4245721

[R30] Eluvathingal T. J., Hasan K. M., Kramer L., Fletcher J. M., & Ewing-Cobbs L. (2007). Quantitative diffusion tensor tractography of association and projection fibers in normally developing children and adolescents. Cerebral Cortex, 17(12), 2760–2768. 17307759 10.1093/cercor/bhm003 PMC2084482

[R31] Fan D., Chaudhari N. N., Rostowsky K. A., Calvillo M., Lee S. K., Chowdhury N. F., Zhang F., O’Donnell L. J., & Irimia A. (2019). Post-Traumatic Cerebral Microhemorrhages and their Effects Upon White Matter Connectivity in the Aging Human Brain. 2019 41st Annual International Conference of the IEEE Engineering in Medicine and Biology Society (EMBC), 198–203. 10.1109/EMBC.2019.8857921 PMC7041304 31945877

[R32] Ge B., Guo L., Zhang T., Zhu D., Li K., Hu X., Han J., & Liu T. (2012). Group-wise consistent fiber clustering based on multimodal connectional and functional profiles. Medical Image Computing and Computer-Assisted Intervention – MICCAI 2012, 15, 485–492. 10.1007/978-3-642-33454-2_60 23286166

[R33] Giedd J. N., Raznahan A., Mills K. L., & Lenroot R. K. (2012). Review: magnetic resonance imaging of male/female differences in human adolescent brain anatomy. Biology of Sex Differences, 3(1), 19. 22908911 10.1186/2042-6410-3-19 PMC3472204

[R34] Glasser M. F., Sotiropoulos S. N., Wilson J. A., Coalson T. S., Fischl B., Andersson J. L., Xu J., Jbabdi S., Webster M., Polimeni J. R., Van Essen D. C., Jenkinson M., & WU-Minn HCP Consortium. (2013). The minimal preprocessing pipelines for the Human Connectome Project. NeuroImage, 80, 105–124. 23668970 10.1016/j.neuroimage.2013.04.127 PMC3720813

[R35] Gong G., He Y., & Evans A. C. (2011). Brain connectivity: gender makes a difference. The Neuroscientist: A Review Journal Bringing Neurobiology, Neurology and Psychiatry, 17(5), 575–591. 21527724 10.1177/1073858410386492

[R36] Gong G., Rosa-Neto P., Carbonell F., Chen Z. J., He Y., & Evans A. C. (2009). Age- and gender-related differences in the cortical anatomical network. The Journal of Neuroscience: The Official Journal of the Society for Neuroscience, 29(50), 15684–15693. 20016083 10.1523/JNEUROSCI.2308-09.2009 PMC2831804

[R37] Gong S., Zhang F., Norton I., Essayed W. I., Unadkat P., Rigolo L., Pasternak O., Rathi Y., Hou L., Golby A. J., & O’Donnell L. J. (2018). Free water modeling of peritumoral edema using multi-fiber tractography: Application to tracking the arcuate fasciculus for neurosurgical planning. PloS One, 13(5), e0197056. 29746544 10.1371/journal.pone.0197056 PMC5944935

[R38] Guevara M., Román C., Houenou J., Duclap D., Poupon C., Mangin J. F., & Guevara P. (2017). Reproducibility of superficial white matter tracts using diffusion-weighted imaging tractography. NeuroImage, 147, 703–725. 28034765 10.1016/j.neuroimage.2016.11.066

[R39] Hagmann P., Kurant M., Gigandet X., Thiran P., Wedeen V. J., Meuli R., & Thiran J.-P. (2007). Mapping human whole-brain structural networks with diffusion MRI. PloS One, 2(7), e597. 17611629 10.1371/journal.pone.0000597 PMC1895920

[R40] Halpern D. F. (2000). Sex Differences in Cognitive Abilities: 3rd Edition. Taylor & Francis.

[R41] Hasan K. M., Iftikhar A., Kamali A., Kramer L. A., Ashtari M., Cirino P. T., Papanicolaou A. C., Fletcher J. M., & Ewing-Cobbs L. (2009). Development and aging of the healthy human brain uncinate fasciculus across the lifespan using diffusion tensor tractography. Brain Research, 1276, 67–76. 19393229 10.1016/j.brainres.2009.04.025 PMC2693464

[R42] Hau J., Sarubbo S., Perchey G., Crivello F., Zago L., Mellet E., Jobard G., Joliot M., Mazoyer B. M., Tzourio-Mazoyer N., & Petit L. (2016). Cortical Terminations of the Inferior Fronto-Occipital and Uncinate Fasciculi: Anatomical Stem-Based Virtual Dissection. Frontiers in Neuroanatomy, 10, 58. 27252628 10.3389/fnana.2016.00058 PMC4877506

[R43] He J., Zhang F., Pan Y., Feng Y., Rushmore J., Torio E., Rathi Y., Makris N., Kikinis R., Golby A. J., & O’Donnell L. J. (2023). Reconstructing the somatotopic organization of the corticospinal tract remains a challenge for modern tractography methods. Human Brain Mapping, 44(17), 6055–6073. 37792280 10.1002/hbm.26497 PMC10619402

[R44] Herlin B., Uszynski I., Chauvel M., Dupont S., & Poupon C. (2024). Sex-related variability of white matter tracts in the whole HCP cohort. Brain Structure & Function, 229(7), 1713–1735. 39012482 10.1007/s00429-024-02833-0 PMC11374878

[R45] Hervé P.-Y., Leonard G., Perron M., Pike B., Pitiot A., Richer L., Veillette S., Pausova Z., & Paus T. (2009). Handedness, motor skills and maturation of the corticospinal tract in the adolescent brain. Human Brain Mapping, 30(10), 3151–3162. 19235881 10.1002/hbm.20734 PMC6870659

[R46] Hosoki M., Bruckert L., Borchers L. R., Marchman V. A., Travis K. E., & Feldman H. M. (2023). Associations of behavioral problems and white matter properties of the cerebellar peduncles in boys and girls born full term and preterm. Cerebellum (London, England), 22(2), 163–172. 35138604 10.1007/s12311-022-01375-7 PMC9360188

[R47] Hotelling H. (1936). Relations Between Two Sets of Variates. Biometrika, 28(3/4), 321–377.

[R48] Hu Y., Xu Q., Li K., Zhu H., Qi R., Zhang Z., & Lu G. (2013). Gender differences of brain glucose metabolic networks revealed by FDG-PET: evidence from a large cohort of 400 young adults. PloS One, 8(12), e83821. 24358312 10.1371/journal.pone.0083821 PMC3866135

[R49] Ingalhalikar M., Smith A., Parker D., Satterthwaite T. D., Elliott M. A., Ruparel K., Hakonarson H., Gur R. E., Gur R. C., & Verma R. (2014). Sex differences in the structural connectome of the human brain. Proceedings of the National Academy of Sciences of the United States of America, 111(2), 823–828. 24297904 10.1073/pnas.1316909110 PMC3896179

[R50] Irimia A., Fan D., Chaudhari N. N., Ngo V., Zhang F., Joshi S. H., & O’Donnell L. J. (2020). Mapping cerebral connectivity changes after mild traumatic brain injury in older adults using diffusion tensor imaging and Riemannian matching of elastic curves. 2020 IEEE 17th International Symposium on Biomedical Imaging (ISBI), 1690–1693.

[R51] Jahanshad N., Aganj I., Lenglet C., Joshi A., Jin Y., Barysheva M., McMahon K. L., de Zubicaray G. I., Martin N. G., Wright M. J., Toga A. W., Sapiro G., & Thompson P. M. (2011). Sex differences in the human connectome: 4-Tesla high angular resolution diffusion imaging (HARDI) tractography in 234 young adult twins. 2011 IEEE International Symposium on Biomedical Imaging: From Nano to Macro, 939–943.

[R52] Jeurissen B., Leemans A., Tournier J.-D., Jones D. K., & Sijbers J. (2013). Investigating the prevalence of complex fiber configurations in white matter tissue with diffusion magnetic resonance imaging. Human Brain Mapping, 34(11), 2747–2766. 22611035 10.1002/hbm.22099 PMC6870534

[R53] Jin Y., Shi Y., Zhan L., Gutman B. A., de Zubicaray G. I., McMahon K. L., Wright M. J., Toga A. W., & Thompson P. M. (2014). Automatic clustering of white matter fibers in brain diffusion MRI with an application to genetics. NeuroImage, 100, 75–90. 24821529 10.1016/j.neuroimage.2014.04.048 PMC4255631

[R54] Jones D. K., & Cercignani M. (2010). Twenty-five pitfalls in the analysis of diffusion MRI data. NMR in Biomedicine, 23(7), 803–820. 20886566 10.1002/nbm.1543

[R55] Jörntell H. (2017). Cerebellar physiology: links between microcircuitry properties and sensorimotor functions. The Journal of Physiology, 595(1), 11–27. 27388692 10.1113/JP272769 PMC5199730

[R56] Jung M., Mody M., Fujioka T., Kimura Y., Okazawa H., & Kosaka H. (2019). Sex Differences in White Matter Pathways Related to Language Ability. Frontiers in Neuroscience, 13, 898. 31555075 10.3389/fnins.2019.00898 PMC6723765

[R57] Kai J., & Khan A. R. (2019). Assessing white matter pathway reproducibility from human whole-brain tractography clustering. bioRxiv, 833095.

[R58] Kanaan R. A., Allin M., Picchioni M., Barker G. J., Daly E., Shergill S. S., Woolley J., & McGuire P. K. (2012). Gender differences in white matter microstructure. PloS One, 7(6), e38272. 22701619 10.1371/journal.pone.0038272 PMC3368921

[R59] Kanaan R. A., Chaddock C., Allin M., Picchioni M. M., Daly E., Shergill S. S., & McGuire P. K. (2014). Gender influence on white matter microstructure: a tract-based spatial statistics analysis. PloS One, 9(3), e91109. 24603769 10.1371/journal.pone.0091109 PMC3946347

[R60] Kang X., Adamson M. M., & Yoon B. C. (2025). Sex differences in structural and functional connectivity in healthy young adults from the Amsterdam Open MRI Collection. Brain Structure & Function, 230(6), 98. 40522500 10.1007/s00429-025-02965-x

[R61] Kitamura S., Morikawa M., Kiuchi K., Taoka T., Fukusumi M., Kichikawa K., & Kishimoto T. (2011). Asymmetry, sex differences and age-related changes in the white matter in the healthy elderly: a tract-based study. BMC Research Notes, 4, 378. 21970546 10.1186/1756-0500-4-378 PMC3205060

[R62] Lebel C., Caverhill-Godkewitsch S., & Beaulieu C. (2010). Age-related regional variations of the corpus callosum identified by diffusion tensor tractography. NeuroImage, 52(1), 20–31. 20362683 10.1016/j.neuroimage.2010.03.072

[R63] Lei X., Han Z., Chen C., Bai L., Xue G., & Dong Q. (2016). Sex Differences in Fiber Connection between the Striatum and Subcortical and Cortical Regions. Frontiers in Computational Neuroscience, 10, 100. 27721750 10.3389/fncom.2016.00100 PMC5034007

[R64] Levitt J. J., Zhang F., Vangel M., Nestor P. G., Rathi Y., Cetin-Karayumak S., Kubicki M., Coleman M. J., Lewandowski K. E., Holt D. J., Keshavan M., Bouix S., Öngür D., Breier A., Shenton M. E., & O’Donnell L. J. (2023). The organization of frontostriatal brain wiring in non-affective early psychosis compared with healthy subjects using a novel diffusion imaging fiber cluster analysis. Molecular Psychiatry, 28(6), 2301–2311. 37173451 10.1038/s41380-023-02031-0 PMC11971472

[R65] Liao R., Ning L., Chen Z., Rigolo L., Gong S., Pasternak O., Golby A. J., Rathi Y., & O’Donnell L. J. (2017). Performance of unscented Kalman filter tractography in edema: Analysis of the two-tensor model. NeuroImage. Clinical, 15, 819–831. 28725549 10.1016/j.nicl.2017.06.027 PMC5506885

[R66] Liégeois R., Li J., Kong R., Orban C., Van De Ville D., Ge T., Sabuncu M. R., & Yeo B. T. T. (2019). Resting brain dynamics at different timescales capture distinct aspects of human behavior. Nature Communications, 10(1), 2317. 10.1038/s41467-019-10317-7 PMC6534566 31127095

[R67] Liu F., Vidarsson L., Winter J. D., Tran H., & Kassner A. (2010). Sex differences in the human corpus callosum microstructure: a combined T2 myelin-water and diffusion tensor magnetic resonance imaging study. Brain Research, 1343, 37–45. 20435024 10.1016/j.brainres.2010.04.064

[R68] Liu R., Li M., & Dunson D. B. (2023). PPA: Principal parcellation analysis for brain connectomes and multiple traits. NeuroImage, 276(120214), 120214. 37286151 10.1016/j.neuroimage.2023.120214

[R69] Liu Y., Metens T., Absil J., De Maertelaer V., Balériaux D., David P., Denolin V., Van Overmeire B., Avni F., Van Bogaert P., & Aeby A. (2011). Gender Differences in Language and Motor-Related Fibers in a Population of Healthy Preterm Neonates at Term-Equivalent Age: A Diffusion Tensor and Probabilistic Tractography Study. American Journal of Neuroradiology, 32(11), 2011–2016. 21940804 10.3174/ajnr.A2690 PMC7964391

[R70] Li Y., Zhang W., Wu Y., Yin L., Zhu C., Chen Y., Cetin-Karayumak S., Cho K. I. K., Zekelman L. R., Rushmore J., Rathi Y., Makris N., O’Donnell L. J., & Zhang F. (2024). A diffusion MRI tractography atlas for concurrent white matter mapping across Eastern and Western populations. Scientific Data, 11(1), 1–14. 39019877 10.1038/s41597-024-03624-2 PMC11255335

[R71] Lo Y., Chen Y., Liu D., Liu W., Zekelman L., Rushmore J., Zhang F., Rathi Y., Makris N., Golby A. J., Cai W., & O’Donnell L. J. (2025). The shape of the brain’s connections is predictive of cognitive performance: an explainable machine learning study. Human Brain Mapping, 46(5), e70166. 40143640 10.1002/hbm.70166 PMC11947434

[R72] Madhavan K. M., McQueeny T., Howe S. R., Shear P., & Szaflarski J. (2014). Superior longitudinal fasciculus and language functioning in healthy aging. Brain Research, 1562, 11–22. 24680744 10.1016/j.brainres.2014.03.012 PMC4049076

[R73] Maier-Hein K. H., Neher P. F., Houde J.-C., Côté M.-A., Garyfallidis E., Zhong J., Chamberland M., Yeh F.-C., Lin Y.-C., Ji Q., Reddick W. E., Glass J. O., Chen D. Q., Feng Y., Gao C., Wu Y., Ma J., Renjie H., Li Q., … Descoteaux M. (2017). The challenge of mapping the human connectome based on diffusion tractography. Nature Communications, 8(1), 1349. 10.1038/s41467-017-01285-x PMC5677006 29116093

[R74] Makris N., Meyer J. W., Bates J. F., Yeterian E. H., Kennedy D. N., & Caviness V. S. (1999). MRI-Based topographic parcellation of human cerebral white matter and nuclei II. Rationale and applications with systematics of cerebral connectivity. NeuroImage, 9(1), 18–45. 9918726 10.1006/nimg.1998.0384

[R75] Malcolm J. G., Shenton M. E., & Rathi Y. (2010). Filtered multitensor tractography. IEEE Transactions on Medical Imaging, 29(9), 1664–1675. 20805043 10.1109/TMI.2010.2048121 PMC3045040

[R76] Menzler K., Belke M., Wehrmann E., Krakow K., Lengler U., Jansen A., Hamer H. M., Oertel W. H., Rosenow F., & Knake S. (2011). Men and women are different: diffusion tensor imaging reveals sexual dimorphism in the microstructure of the thalamus, corpus callosum and cingulum. NeuroImage, 54(4), 2557–2562. 21087671 10.1016/j.neuroimage.2010.11.029

[R77] Mormina E., Petracca M., Bommarito G., Piaggio N., Cocozza S., & Inglese M. (2017). Cerebellum and neurodegenerative diseases: Beyond conventional magnetic resonance imaging. World Journal of Radiology, 9(10), 371–388. 29104740 10.4329/wjr.v9.i10.371 PMC5661166

[R78] Morris L. S., Kundu P., Dowell N., Mechelmans D. J., Favre P., Irvine M. A., Robbins T. W., Daw N., Bullmore E. T., Harrison N. A., & Voon V. (2016). Fronto-striatal organization: Defining functional and microstructural substrates of behavioural flexibility. Cortex; a Journal Devoted to the Study of the Nervous System and Behavior, 74, 118–133. 26673945 10.1016/j.cortex.2015.11.004 PMC4729321

[R79] Moyer D., Gutman B. A., Faskowitz J., Jahanshad N., & Thompson P. M. (2017). Continuous representations of brain connectivity using spatial point processes. Medical Image Analysis, 41, 32–39. 28487128 10.1016/j.media.2017.04.013 PMC5559296

[R80] Nichols T. E., & Holmes A. P. (2002). Nonparametric permutation tests for functional neuroimaging: a primer with examples. Human Brain Mapping, 15(1), 1–25. 11747097 10.1002/hbm.1058 PMC6871862

[R81] Nimsky C. (2014). Fiber tracking—we should move beyond diffusion tensor imaging. World Neurosurgery, 82(1), 35–36. 24007693 10.1016/j.wneu.2013.08.035

[R82] Nordahl C. W., Iosif A.-M., Young G. S., Perry L. M., Dougherty R., Lee A., Li D., Buonocore M. H., Simon T., Rogers S., Wandell B., & Amaral D. G. (2015). Sex differences in the corpus callosum in preschool-aged children with autism spectrum disorder. Molecular Autism, 6(1), 26. 25973163 10.1186/s13229-015-0005-4 PMC4429319

[R83] O’Donnell L. J., Golby A. J., & Westin C.-F. (2013). Fiber clustering versus the parcellation-based connectome. NeuroImage, 80, 283–289. 23631987 10.1016/j.neuroimage.2013.04.066 PMC3731058

[R84] O’Donnell L. J., Suter Y., Rigolo L., Kahali P., Zhang F., Norton I., Albi A., Olubiyi O., Meola A., Essayed W. I., Unadkat P., Ciris P. A., Wells W. M. 3rd, Rathi Y., Westin C.-F., & Golby A. J. (2017). Automated white matter fiber tract identification in patients with brain tumors. NeuroImage. Clinical, 13, 138–153. 27981029 10.1016/j.nicl.2016.11.023 PMC5144756

[R85] O’Donnell L. J., Wells W. M. III, Golby A. J., & Westin C.-F. (2012). Unbiased Groupwise Registration of White Matter Tractography. Medical Image Computing and Computer-Assisted Intervention – MICCAI 2012, 123–130. 10.1007/978-3-642-33454-2_16 PMC3638882 23286122

[R86] O’Donnell L. J., & Westin C.-F. (2007). Automatic tractography segmentation using a high-dimensional white matter atlas. IEEE Transactions on Medical Imaging, 26(11), 1562–1575. 18041271 10.1109/TMI.2007.906785

[R87] Oh J. S., Song I. C., Lee J. S., Kang H., Park K. S., Kang E., & Lee D. S. (2007). Tractography-guided statistics (TGIS) in diffusion tensor imaging for the detection of gender difference of fiber integrity in the midsagittal and parasagittal corpora callosa. NeuroImage, 36(3), 606–616. 17481923 10.1016/j.neuroimage.2007.03.020

[R88] Peper J. S., Hulshoff Pol H. E., Crone E. A., & van Honk J. (2011). Sex steroids and brain structure in pubertal boys and girls: a mini-review of neuroimaging studies. Neuroscience, 191, 28–37. 21335066 10.1016/j.neuroscience.2011.02.014

[R89] Phillips O. R., Clark K. A., Luders E., Azhir R., Joshi S. H., Woods R. P., Mazziotta J. C., Toga A. W., & Narr K. L. (2013). Superficial white matter: effects of age, sex, and hemisphere. Brain Connectivity, 3(2), 146–159. 23461767 10.1089/brain.2012.0111 PMC3634148

[R90] Pujol S., Wells W., Pierpaoli C., Brun C., Gee J., Cheng G., Vemuri B., Commowick O., Prima S., Stamm A., Goubran M., Khan A., Peters T., Neher P., Maier-Hein K. H., Shi Y., Tristan-Vega A., Veni G., Whitaker R., … Kikinis R. (2015). The DTI Challenge: Toward Standardized Evaluation of Diffusion Tensor Imaging Tractography for Neurosurgery. Journal of Neuroimaging: Official Journal of the American Society of Neuroimaging, 25(6), 875–882. 26259925 10.1111/jon.12283 PMC4641305

[R91] Raffelt D. A., Smith R. E., Ridgway G. R., Tournier J.-D., Vaughan D. N., Rose S., Henderson R., & Connelly A. (2015). Connectivity-based fixel enhancement: Whole-brain statistical analysis of diffusion MRI measures in the presence of crossing fibres. NeuroImage, 117, 40–55. 26004503 10.1016/j.neuroimage.2015.05.039 PMC4528070

[R92] Reddy C. P., & Rathi Y. (2016). Joint Multi-Fiber NODDI Parameter Estimation and Tractography Using the Unscented Information Filter. Frontiers in Neuroscience, 10, 166. 27147956 10.3389/fnins.2016.00166 PMC4837399

[R93] Ritchie S. J., Cox S. R., Shen X., Lombardo M. V., Reus L. M., Alloza C., Harris M. A., Alderson H. L., Hunter S., Neilson E., Liewald D. C. M., Auyeung B., Whalley H. C., Lawrie S. M., Gale C. R., Bastin M. E., McIntosh A. M., & Deary I. J. (2018). Sex Differences in the Adult Human Brain: Evidence from 5216 UK Biobank Participants. Cerebral Cortex, 28(8), 2959–2975. 29771288 10.1093/cercor/bhy109 PMC6041980

[R94] Ruigrok A. N. V., Salimi-Khorshidi G., Lai M.-C., Baron-Cohen S., Lombardo M. V., Tait R. J., & Suckling J. (2014). A meta-analysis of sex differences in human brain structure. Neuroscience and Biobehavioral Reviews, 39, 34–50. 24374381 10.1016/j.neubiorev.2013.12.004 PMC3969295

[R95] Sen B., & Parhi K. K. (2019). Predicting Male vs. Female from Task-fMRI Brain Connectivity. 2019 41st Annual International Conference of the IEEE Engineering in Medicine and Biology Society (EMBC), 4089–4092. 10.1109/EMBC.2019.8857236 31946770

[R96] Seunarine K. K., Clayden J. D., Jentschke S., Muñoz M., Cooper J. M., Chadwick M. J., Banks T., Vargha-Khadem F., & Clark C. A. (2016). Sexual Dimorphism in White Matter Developmental Trajectories Using Tract-Based Spatial Statistics. Brain Connectivity, 6(1), 37–47. 26446207 10.1089/brain.2015.0340 PMC4744889

[R97] Shiino A., Chen Y.-W., Tanigaki K., Yamada A., Vigers P., Watanabe T., Tooyama I., & Akiguchi I. (2017). Sex-related difference in human white matter volumes studied: Inspection of the corpus callosum and other white matter by VBM. Scientific Reports, 7, 39818. 28045130 10.1038/srep39818 PMC5206615

[R98] Shin Y.-W., Kim D. J., Ha T. H., Park H.-J., Moon W.-J., Chung E. C., Lee J. M., Kim I. Y., Kim S. I., & Kwon J. S. (2005). Sex differences in the human corpus callosum: diffusion tensor imaging study. Neuroreport, 16(8), 795–798. 15891572 10.1097/00001756-200505310-00003

[R99] Smith S. M., & Nichols T. E. (2009). Threshold-free cluster enhancement: addressing problems of smoothing, threshold dependence and localisation in cluster inference. NeuroImage, 44(1), 83–98. 18501637 10.1016/j.neuroimage.2008.03.061

[R100] Smith S. M., Nichols T. E., Vidaurre D., Winkler A. M., Behrens T. E. J., Glasser M. F., Ugurbil K., Barch D. M., Van Essen D. C., & Miller K. L. (2015). A positive-negative mode of population covariation links brain connectivity, demographics and behavior. Nature Neuroscience, 18(11), 1565–1567. 26414616 10.1038/nn.4125 PMC4625579

[R101] Sotiropoulos S. N., & Zalesky A. (2019). Building connectomes using diffusion MRI: why, how and but. NMR in Biomedicine, 32(4), e3752. 28654718 10.1002/nbm.3752 PMC6491971

[R102] Stojanovski S., Felsky D., Viviano J. D., Shahab S., Bangali R., Burton C. L., Devenyi G. A., O’Donnell L. J., Szatmari P., Chakravarty M. M., Ameis S., Schachar R., Voineskos A. N., & Wheeler A. L. (2019). Polygenic Risk and Neural Substrates of Attention-Deficit/Hyperactivity Disorder Symptoms in Youths With a History of Mild Traumatic Brain Injury. Biological Psychiatry, 85(5), 408–416. 30119875 10.1016/j.biopsych.2018.06.024 PMC6330150

[R103] Sydnor V. J., Rivas-Grajales A. M., Lyall A. E., Zhang F., Bouix S., Karmacharya S., Shenton M. E., Westin C.-F., Makris N., Wassermann D., O’Donnell L. J., & Kubicki M. (2018). A comparison of three fiber tract delineation methods and their impact on white matter analysis. NeuroImage, 178, 318–331. 29787865 10.1016/j.neuroimage.2018.05.044 PMC6481642

[R104] Szalkai B., & Grolmusz V. (2018). Human Sexual Dimorphism of the Relative Cerebral Area Volumes in the Data of the Human Connectome Project. European Journal of Anatomy, 22(3), 221–225.

[R105] Szeszko P. R., Vogel J., Ashtari M., Malhotra A. K., Bates J., Kane J. M., Bilder R. M., Frevert T., & Lim K. (2003). Sex differences in frontal lobe white matter microstructure: a DTI study. Neuroreport, 14(18), 2469–2473. 14663212 10.1097/00001756-200312190-00035

[R106] Takao H., Hayashi N., & Ohtomo K. (2014). Sex dimorphism in the white matter: fractional anisotropy and brain size. Journal of Magnetic Resonance Imaging: JMRI, 39(4), 917–923. 24123288 10.1002/jmri.24225

[R107] Thiebaut de Schotten M., Ffytche D. H., Bizzi A., Dell’Acqua F., Allin M., Walshe M., Murray R., Williams S. C., Murphy D. G. M., & Catani M. (2011). Atlasing location, asymmetry and inter-subject variability of white matter tracts in the human brain with MR diffusion tractography. NeuroImage, 54(1), 49–59. 20682348 10.1016/j.neuroimage.2010.07.055

[R108] Tomasi D., & Volkow N. D. (2012). Gender differences in brain functional connectivity density. Human Brain Mapping, 33(4), 849–860. 21425398 10.1002/hbm.21252 PMC3250567

[R109] Travis K. E., Leitner Y., Feldman H. M., & Ben-Shachar M. (2015). Cerebellar white matter pathways are associated with reading skills in children and adolescents. Human Brain Mapping, 36(4), 1536–1553. 25504986 10.1002/hbm.22721 PMC4374012

[R110] Tunç B., Ingalhalikar M., Parker D., Lecoeur J., Singh N., Wolf R. L., Macyszyn L., Brem S., & Verma R. (2016). Individualized Map of White Matter Pathways: Connectivity-Based Paradigm for Neurosurgical Planning. Neurosurgery, 79(4), 568–577. 26678299 10.1227/NEU.0000000000001183 PMC4911597

[R111] Tyan Y.-S., Liao J.-R., Shen C.-Y., Lin Y.-C., & Weng J.-C. (2017). Gender differences in the structural connectome of the teenage brain revealed by generalized q-sampling MRI. NeuroImage. Clinical, 15, 376–382. 28580294 10.1016/j.nicl.2017.05.014 PMC5447512

[R112] Van Essen D. C., Smith S. M., Barch D. M., Behrens T. E. J., Yacoub E., Ugurbil K., & WU-Minn HCP Consortium. (2013). The WU-Minn Human Connectome Project: an overview. NeuroImage, 80, 62–79. 23684880 10.1016/j.neuroimage.2013.05.041 PMC3724347

[R113] Van Essen D. C., Ugurbil K., Auerbach E., Barch D., Behrens T. E. J., Bucholz R., Chang A., Chen L., Corbetta M., Curtiss S. W., Della Penna S., Feinberg D., Glasser M. F., Harel N., Heath A. C., Larson-Prior L., Marcus D., Michalareas G., Moeller S., … WU-Minn HCP Consortium. (2012). The Human Connectome Project: a data acquisition perspective. NeuroImage, 62(4), 2222–2231. 22366334 10.1016/j.neuroimage.2012.02.018 PMC3606888

[R114] Wang G., Erpelding N., & Davis K. D. (2014). Sex differences in connectivity of the subgenual anterior cingulate cortex. Pain, 155(4), 755–763. 24434729 10.1016/j.pain.2014.01.005

[R115] Wang Y., Adamson C., Yuan W., Altaye M., Rajagopal A., Byars A. W., & Holland S. K. (2012). Sex differences in white matter development during adolescence: a DTI study. Brain Research, 1478, 1–15. 22954903 10.1016/j.brainres.2012.08.038 PMC3592389

[R116] Wassermann D., Makris N., Rathi Y., Shenton M., Kikinis R., Kubicki M., & Westin C.-F. (2016). The white matter query language: a novel approach for describing human white matter anatomy. Brain Structure & Function, 221(9), 4705–4721. 26754839 10.1007/s00429-015-1179-4 PMC4940319

[R117] Weis S., Patil K. R., Hoffstaedter F., Nostro A., Yeo B. T. T., & Eickhoff S. B. (2019). Sex Classification by Resting State Brain Connectivity. Cerebral Cortex, 30(2), 824–835. 10.1093/cercor/bhz129 PMC7444737 31251328

[R118] Westerhausen R., Walter C., Kreuder F., Wittling R. A., Schweiger E., & Wittling W. (2003). The influence of handedness and gender on the microstructure of the human corpus callosum: a diffusion-tensor magnetic resonance imaging study. Neuroscience Letters, 351(2), 99–102. 14583391 10.1016/j.neulet.2003.07.011

[R119] Wheelock M. D., Goodman A. M., Harnett N. G., Wood K. H., Mrug S., Granger D. A., & Knight D. C. (2021). Sex-related differences in stress reactivity and cingulum white matter. Neuroscience, 459, 118–128. 33588003 10.1016/j.neuroscience.2021.02.014 PMC7965343

[R120] Wu Y., Zhang F., Makris N., Ning Y., Norton I., She S., Peng H., Rathi Y., Feng Y., Wu H., & O’Donnell L. J. (2018). Investigation into local white matter abnormality in emotional processing and sensorimotor areas using an automatically annotated fiber clustering in major depressive disorder. NeuroImage, 181, 16–29. 29890329 10.1016/j.neuroimage.2018.06.019 PMC6415925

[R121] Xie G., Zhang F., Leung L., Mooney M. A., Epprecht L., Norton I., Rathi Y., Kikinis R., Al-Mefty O., Makris N., Golby A. J., & O’Donnell L. J. (2020). Anatomical assessment of trigeminal nerve tractography using diffusion MRI: A comparison of acquisition b-values and single- and multi-fiber tracking strategies. NeuroImage. Clinical, 25, 102160. 31954337 10.1016/j.nicl.2019.102160 PMC6962690

[R122] Xin J., Zhang Y., Tang Y., & Yang Y. (2019). Brain Differences Between Men and Women: Evidence From Deep Learning. Frontiers in Neuroscience, 13, 185. 30906246 10.3389/fnins.2019.00185 PMC6418873

[R123] Yamada K., Sakai K., Akazawa K., Yuen S., & Nishimura T. (2009). MR Tractography: A Review of Its Clinical Applications. Magnetic Resonance in Medical Sciences: MRMS: An Official Journal of Japan Society of Magnetic Resonance in Medicine, 8(4), 165–174. 20035125 10.2463/mrms.8.165

[R124] Yan C., Gong G., Wang J., Wang D., Liu D., Zhu C., Chen Z. J., Evans A., Zang Y., & He Y. (2011). Sex- and Brain Size–Related Small-World Structural Cortical Networks in Young Adults: A DTI Tractography Study. Cerebral Cortex, 21(2), 449–458. 20562318 10.1093/cercor/bhq111

[R125] Zekelman L. R., Zhang F., Makris N., He J., Chen Y., Xue T., Liera D., Drane D. L., Rathi Y., Golby A. J., & O’Donnell L. J. (2022). White matter association tracts underlying language and theory of mind: An investigation of 809 brains from the Human Connectome Project. NeuroImage, 246, 118739. 34856375 10.1016/j.neuroimage.2021.118739 PMC8862285

[R126] Zhang C., Dougherty C. C., Baum S. A., White T., & Michael A. M. (2018). Functional connectivity predicts gender: Evidence for gender differences in resting brain connectivity. Human Brain Mapping, 39(4), 1765–1776. 29322586 10.1002/hbm.23950 PMC6866578

[R127] Zhang F., Daducci A., He Y., Schiavi S., Seguin C., Smith R. E., Yeh C.-H., Zhao T., & O’Donnell L. J. (2022). Quantitative mapping of the brain’s structural connectivity using diffusion MRI tractography: A review. NeuroImage, 249, 118870. 34979249 10.1016/j.neuroimage.2021.118870 PMC9257891

[R128] Zhang F., Noh T., Juvekar P., Frisken S. F., Rigolo L., Norton I., Kapur T., Pujol S., Iii W. W., Yarmarkovich A., Kindlmann G., Wassermann D., Estepar R. S. J., Rathi Y., Kikinis R., Johnson H. J., Westin C.-F., Pieper S., Golby A. J., & O’Donnell A. L. J. (2020). SlicerDMRI: Diffusion MRI and Tractography Research Software for Brain Cancer Surgery Planning and Visualization. JCO Clinical Cancer Informatics, 4, 299–309. 32216636 10.1200/CCI.19.00141 PMC7113081

[R129] Zhang F., Norton I., Cai W., Song Y., Wells W. M., & O’Donnell L. J. (2017). Comparison between two white matter segmentation strategies: An investigation into white matter segmentation consistency. 2017 IEEE 14th International Symposium on Biomedical Imaging (ISBI 2017), 796–799.

[R130] Zhang F., Savadjiev P., Cai W., Song Y., Rathi Y., Tunç B., Parker D., Kapur T., Schultz R. T., Makris N., Verma R., & O’Donnell L. J. (2018). Whole brain white matter connectivity analysis using machine learning: An application to autism. NeuroImage, 172, 826–837. 29079524 10.1016/j.neuroimage.2017.10.029 PMC5910272

[R131] Zhang F., Vangel M., Kubicki M., Shenton M. E., O’Donnell L. J., & Levitt A. J. J. (2020). Assessing brain wiring patterns using tractography fiber clustering to study sex differences of the frontostriatal white matter connections. ISMRM, 4490.

[R132] Zhang F., Wu W., Ning L., McAnulty G., Waber D., Gagoski B., Sarill K., Hamoda H. M., Song Y., Cai W., Rathi Y., & O’Donnell L. J. (2018). Suprathreshold fiber cluster statistics: Leveraging white matter geometry to enhance tractography statistical analysis. NeuroImage, 171, 341–354. 29337279 10.1016/j.neuroimage.2018.01.006 PMC5857470

[R133] Zhang F., Wu Y., Norton I., Rathi Y., Golby A. J., & O’Donnell L. J. (2019). Test-retest reproducibility of white matter parcellation using diffusion MRI tractography fiber clustering. Human Brain Mapping, 40(10), 3041–3057. 30875144 10.1002/hbm.24579 PMC6548665

[R134] Zhang F., Wu Y., Norton I., Rigolo L., Rathi Y., Makris N., & O’Donnell L. J. (2018). An anatomically curated fiber clustering white matter atlas for consistent white matter tract parcellation across the lifespan. NeuroImage, 179, 429–447. 29920375 10.1016/j.neuroimage.2018.06.027 PMC6080311

[R135] Zhang F., Xie G., Leung L., Mooney M. A., Epprecht L., Norton I., Rathi Y., Kikinis R., Al-Mefty O., Makris N., Golby A. J., & O’Donnell L. J. (2020). Creation of a novel trigeminal tractography atlas for automated trigeminal nerve identification. NeuroImage, 117063. 32574805 10.1016/j.neuroimage.2020.117063 PMC7572753

[R136] Zhang J., Gao J., Shi H., Huang B., Wang X., Situ W., Cai W., Yi J., Zhu X., & Yao S. (2014). Sex Differences of Uncinate Fasciculus Structural Connectivity in Individuals with Conduct Disorder. BioMed Research International, 2014, 1–9. 10.1155/2014/673165 PMC4009134 24829912

[R137] Ziyan U., Sabuncu M. R., Grimson W. E. L., & Westin C.-F. (2009). Consistency Clustering: A Robust Algorithm for Group-wise Registration, Segmentation and Automatic Atlas Construction in Diffusion MRI. International Journal of Computer Vision, 85(3), 279–290. 20442792 10.1007/s11263-009-0217-1 PMC2862392

